# Chikungunya Fever, Hong Kong

**DOI:** 10.3201/eid1211.060574

**Published:** 2006-11

**Authors:** Nelson Lee, Chun K. Wong, Wai Y. Lam, Ann Wong, Wilina Lim, Christopher W.K. Lam, Clive S. Cockram, Joseph J.Y. Sung, Paul K.S. Chan, Julian W. Tang

**Affiliations:** * The Chinese University of Hong Kong Prince of Wales Hospital, Shatin, New Territories, Hong Kong Special Administrative Region, People's Republic of China;; † Department of Health, Kowloon, Hong Kong Special Administrative Region, People's Republic of China

**Keywords:** Chikungunya, mosquitoborne, arbovirus, viremia, arthropathy, phylogenetics, interferon, pathogenesis, cytokine, IP-10/CXCL10, letter

To the Editor: Chikungunya virus disease, caused by a mosquitoborne alphavirus, is endemic to Africa and Southeast Asia. It typically causes an acute febrile illness, with joint pain and a skin rash. Chronic arthropathy may develop (*1**,**2*). No treatment or vaccine is available, and relatively little research has been conducted into its pathogenesis, compared with that of other arboviruses, such as dengue. Recent reports have described a massive outbreak of chikungunya disease occurring on islands in the Indian Ocean, off the east coast of Africa (*1*). Reemergence of chikungunya has also been reported from Indonesia (*2*).

During March 2006, a 66-year-old Chinese man from Hong Kong visited Mauritius where he was bitten by mosquitoes 2 days before returning to Hong Kong. On the return trip, he experienced fever (39°C), severe finger joint and muscle pains, mild headache, and a skin rash, and he sought treatment at the Prince of Wales Hospital (PWH) Infectious Diseases Clinic on the second day of his illness. Physical examination showed a generalized erythematous rash over the trunk and limbs and petechiae over the lower limbs. Mild finger joint stiffness was observed, but no joint swelling. No lymphadenopathy or eschar was detected. Level of C-reactive protein was elevated at 10.4 mg/L. Results of screens for malaria and dengue were negative. Results of other routine assessments were unremarkable. His symptoms subsided gradually within a week.

Serum specimens taken on days 2 and 6 were positive for chikungunya virus RNA by in-house reverse transcription (RT)-PCR at the Public Health Laboratory Service (PHLS) (targeting the nonstructural protein-1 [NSP-1] gene) and PWH laboratory (targeting both NSP-1 and the envelope glycoprotein [E1] gene). An additional serum sample taken on day 8 of illness, received by PHLS only, was also positive for chikungunya RNA. Both laboratories confirmed RT-PCR results by sequencing. At PWH, phylogenetic analysis was performed to determine the likely origin of the virus. In-house immunofluorescent slide serologic assays developed at PHLS found chikungunya immunoglobulin G (IgG) titers of <10, 160, and 320 in the serum samples taken on days 2, 6, and 8 of illness, respectively, and detected chikungunya IgM in the day 8 serum. The acute cytokine immunologic response to this virus was also tested (Appendix).

Sequencing and phylogenetic analysis was consistent with an imported infection, almost certainly originating from the current chikungunya outbreaks in the Indian Ocean. Phylogenetic analyses of the NSP-1 and E1 regions, indicated that this virus is most closely related to previous African rather than Southeast Asian chikungunya viruses (Figure A1, Figure A2). The persistence of viremia up to at least day 8 of illness was unusual. Standard texts state that viremia may be present during the first 2–4 days of illness, with neutralizing antibodies appearing by days 5–7 (*3*).

The most striking finding from the cytokine analysis (Table) is the high level of interferon-γ (IFN-γ)–inducible protein-10 (IP-10/CXCL10), up to 26 and 16 times the upper limit of the normal range at days 2 and 6 after disease onset, respectively. Serum concentrations of interleukin-8 (IL-8), monocyte chemoattractant protein (MCP) 1 (MCP-1) and monokine induced by IFN-γ (MIG/CXCL9) are also elevated in both samples. Notably, serum IFN-γ, tumor necrosis factor-α (TNF-α), and IL-1β, 6, 10, and 12 concentrations remain within normal limits in both samples, although the concentrations at local inflammatory sites (e.g., joints) are unknown. CXCL10 and MCP-1/CCL2 concentrations decreased during clinical recovery. Thus, the cytokine profile demonstrates that the levels of Th1 chemokine CXCL10 was highly elevated and that the levels of chemokines IL-8/CXCL8, CCL2, and CXCL9 were moderately elevated. In contrast, IFN-γ and other inflammatory/Th2 cytokines were not elevated during the illness.

**Table:** Serum cytokine profiles of patient with chikungunya infection, days 2 and 6 of illness*

Serum cytokine profile (ng/L)	Time after onset of illness	
	2 d	6 d
IFN-γ, N<15.6	UD	UD
IL-1β, N<7.2	2.4	1.5
IL-6, N<3.1	UD	1.3
IL-10, N<7.8	1.7	1.7
IL-12 p70, N<7.8	3.6	1.4
TNF-α, N<10.0	UD	UD
CXCL8, N<10.0	45.3	54.3
CXCL10†, N = 232–1,019	26,319	16,156
CCL2†, N = 18–152	445	257
CXCL9†, N = 37–463	1,138	1,605
CCL5, N = 10,349–46,704	24,745	60,671

*N, normal range; IFN, interferon; IL, interleukin; UD, undetected; TNF, tumor necrosis factor. †Both 2 d and 6 d samples above normal range.

Interpretation of the significance of these cytokine results is necessarily speculative. Some comparison can be made with other viral infections. In severe acute respiratory syndrome–associated coronavirus (SARS-CoV) (*4**,**5*) and H5N1 influenza (*6*) infections, very high blood levels of CXCL10 and moderately high CCL2, CXCL9, and CXCL8 concentrations, or their enhanced expressions in vitro, have been reported. In dengue fever, which has similar clinical manifestations as chikungunya fever, only elevated CXCL8, IL-6, IL-10, and TNF-α concentrations have been shown consistently (*7**,**8*), although CXCL10 expression has not been studied.

The function of CXCL10 is to act as a chemoattractant for Th1 cells in the activation of cell-mediated immune response. Its expression can be up-regulated by the Th1 cytokine IFN-γ during acute inflammation. CXCL10 has been implicated in the pathogenesis of SARS-CoV and H5N1 influenza infections, in which persistently high CXCL10 concentrations seem to correlate with disease severity and progression (*4**–**6*). CCL2, CXCL9, and CXCL8, have also been found to have a pathogenic role in H5N1 influenza, SARS-CoV, and dengue infections. Notably, the level of antiviral cytokine IFN-γ was not elevated in our chikungunya case, though admittedly, this is only 1 case. This finding may represent a way that the chikungunya virus evades host defenses and may provide a rationale for the use of IFN as a therapeutic option (*9*). Such IFN therapy has been suggested and tried, experimentally, for SARS-CoV (5) and dengue infections (*10*).

## Figures and Tables

**Figure A1:**
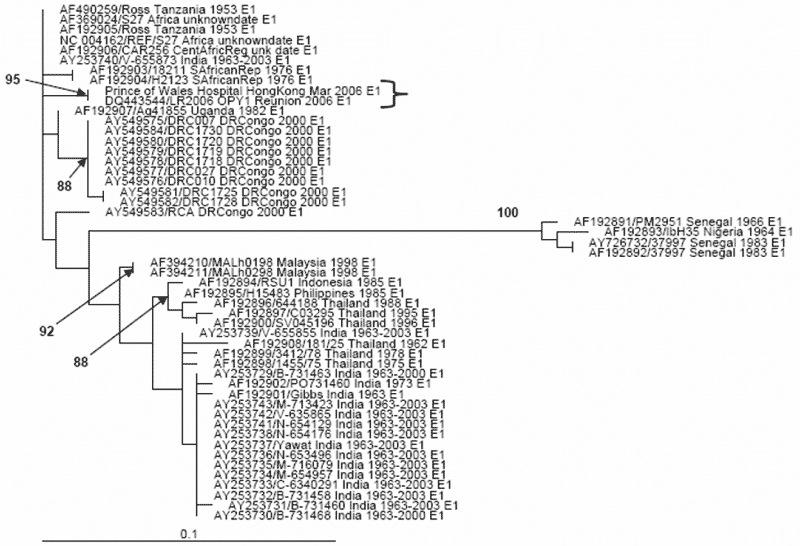
The maximum-likelihood phylogenetic tree for chikungunya envelope glyprotein 1 (E1) sequences (199 bp) constructed by using PAUP* under a Tamura-Nei 1993 model with invariable sites (i.e., TrN+I), as selected by MODELTEST (version 3.7) under the Akaike information criteria. The bracket shows that the PWH patient E1 sequence (DQ489787) clusters most closely with the outbreak La Reunion sequence (LR2006_OPY1_Reunion_2006_E1, DQ443544). Only bootstrap values >70 are shown and considered significant. The tree is rooted against the earliest sequence (AF490259/Ross_Tanzania_1953).

**Figure A2:**
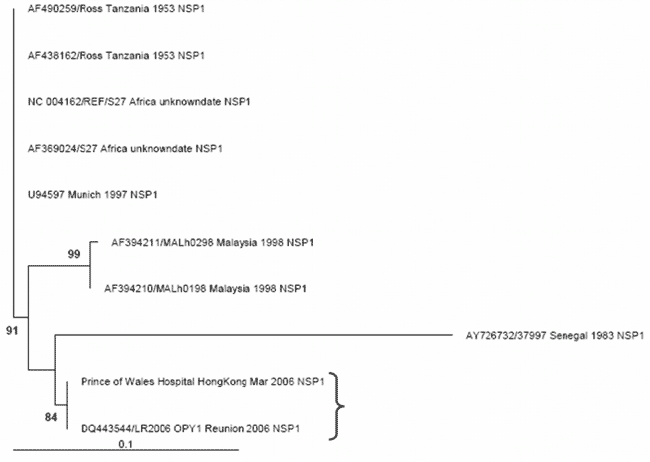
The maximum likelihood phylogenetic tree for chikungunya nonstructural protein 1 (NSP1) sequences (314 bp) constructed using PAUP* under a transitional model with equal base frequencies with a γ-distributed rate of substitution (i.e., TIM+G), as selected by MODELTEST (version 3.7) under the Akaike information criteria. The bracket shows that the PWH patient NSP1 sequence (DQ489788) clusters most closely with the outbreak La Reunion sequence (LR2006_OPY1_Reunion_2006_NSP1, DQ443544). Only bootstrap values >70 are shown and considered significant. The tree is rooted against the earliest sequence (AF490259/Ross_Tanzania_1953).

## Appendix

**Chikungunya Cytokine Profile Determination (Prince of Wales Hospital)**

Serum was prepared from clotted blood by centrifugation (2,000 ×g for 10 min). T helper (Th)1/Th2 cytokines, inflammatory cytokines, and chemokines in serum were measured by cytometric bead array (CBA) by using a 4-color FACSCalibur flow cytometer (Becton Dickinson [BD], San Jose, CA, USA) located in a biosafety level-2 laboratory (1,2). In CBA, 5or 6 bead populations with distinct fluorescence intensities had been coated with capturing antibodies specific for different cytokines or chemokines. These bead populations could be resolved in the fluorescence channels of the flow cytometer. After the beads had been incubated with 50 μL of serum, different cytokines or chemokines in the sample were captured by their corresponding beads. The cytokine/chemokine captured beads were then mixed with phycoerythrin-conjugated detection antibodies to form sandwich complexes. Following incubation, washing, and acquisition of fluorescence data, the results were generated in graphic format by the BD CBA software.

The following concentrations were measured by using the IFN-γ CBA flex set, human inflammatory cytokine and chemokine CBA kits (BD PharMingen, San Diego, CA, USA): Th1/2 cytokines (interferon-γ [IFN-γ] and interleukin 10 [IL-10]), inflammatory cytokines (IL-1β, IL-6, tumor necrosis factor-α, and IL-12p70), and chemokines (IL-8/CXCL8, regulated upon activation normal T cell-expressed and secreted [RANTES/CCL5], monocyte chemoattractant protein-1 [MCP-1/CCL2], IFN-γ-inducible protein-10 [IP-10/CXCL10], and monokine induced by IFN-γ [MIG/CXCL9]. The assay sensitivities of these cytokines and chemokines were 7.1, 3.3, 7.2, 2.5, 3.7, 1.9, 0.2, 1.0, 2.7, 2.8, and 2.5 ng/L, respectively. The coefficients of variation for all cytokine and chemokine assays were <10%. Their respective normal ranges have been derived from measurement of concentrations in >100 healthy subjects (1).

**In-house Chikungunya Immunofluorescent Slide Serologic IgG/IgM assays (Public Health Laboratory Service)**

For measurement of the chikungunya-specific antibody response, an in-house serologic assay was developed. Chikungunya virus obtained from the patient's viremic sera was grown in Vero E6 cells, which were then fixed onto glass slides. Dilutions of patient sera, starting at 1:10, were made up, and 20 μL of each dilution was added to the glass slide and incubated for 45 min at 37°C. They were then washed in phosphate-buffered saline (PBS) for 10 min and blotted dry. Next, 20 μL of a fluorescein-labeled antihuman immunoglobulin M (IgM and polyvalent, from Biosource International, Camarillo, CA, USA) was added to each sample on the slide and incubated for a further 45 min at 37°C. After this, the slides were washed again with PBS for 10 min, blotted dry, mounted with phosphate-buffered glycerol and a coverslip, and then viewed under a UV microscope. The reported immunofluorescent titer was the highest serum dilution showing a positive reaction (apple-green fluorescent cytoplasmic granules).

**Chikungunya RT-PCR and Sequencing (Prince of Wales Hospital)**

Genomic viral RNA was extracted from 140 μL of clinical plasma using the QIAamp ViralRNA MiniKit (Qiagen, Inc., Valencia, CA, USA), according to the manufacturer's instructions, and resuspended in 60 μL of RNase-free water. reverse transcription (RT)-PCR was carried out with SuperScript III One-Step RT-PCR System with Platinum Taq DNA Polymerase kit (Invitrogen, Carlsbad, CA, USA) according to the manufacturer's protocols. In brief, a 50-μL reaction mix, containing 0.5 μM of each forward and reverse primers, and 10 μL of extracted RNA template were used for the RT-PCR. Two previously published primer sets were selected to amplify the nonstructural protein 1 (NSP1) and envelope glycoprotein 1 (E1) genomic regions of the chikungunya viral genome. Primer set 1: (forward: CHIK/NSP1-S 5´-TAGAGCAGGAAATTGATCCC-3´ and reverse: CHIK/NSP1-C 5´- CTTTAATCGCCTGGTGGTAT-3´) (3) was used to amplify the NSP1 region. Primer set 2: (forward: OP16 5´-AGCTGTAAGGTCTTCACCGG-3´ and reverse: OP17 5´- GTATTTTGTTACTATTCAGG-3´) (4) was used to amplify the E1 region. The RT reaction (57°C for 30 min) was followed by 94°C for 2 min and 40 cycles of PCR (94°C for 40 sec, 55°C for 40 sec, and 68°C for 1 min 30 sec, for each cycle) and a final extension at 68°C for 10 min.

The NSP1 (354 bp) and E1 (1.2 kb) PCR products were purified from 1.5% agarose gels by using the Wizard SV Gel and PCR Clean-Up System (Promega, Madison, WI, USA). Sequencing reactions were undertaken with the BigDye Terminator version 3.1 Cycle Sequencing Kits (Applied Biosystems, Foster City, CA, USA). The RT-PCR primer sets were also used for the sequencing reactions. Sequencing was carried out with an automatic sequence analyzer (ABI 3130 Genetic Analyzer, Applied Biosystems) following the manufacturer's protocol. Nucleotide sequences were aligned by using the ClustalX 1.83 software (5). The NSP1 and E1 sequences have been submitted to the National Center for Biotechnology's GenBank under accession nos. DQ489787 (E1) and DQ489788 (NSP1) (available from http://www.ncbi.nlm.nih.gov/entrez/query.fcgi?db=Nucleotide&itool=toolbar).

For phylogenetic analysis, existing NSP1 and E1 sequences were downloaded from GenBank and aligned with ClustalX version 1.83 (5) and edited with Bioedit version 7.0.4.1 (available from http://www.mbio.ncsu.edu/BioEdit/bioedit.html). Many of the NSP1 and E1 sequences downloaded from GenBank were quite short, and we decided to include as many sequences as possible in the final trees, to show the diverse origin of the sequences. Therefore, after editing, the lengths of the sequences used in the final tree construction were 314 bp and 199 bp for NSP1 and E1, respectively. Phylogenetic tree construction was performed with PAUP* version 4.0b10 (6) by using a maximum likelihood (ML) approach, under an optimum model of evolution as determined by MODELTEST version 3.7 (7). Optimal trees were searched for by using a tree-bisection-reconnection heuristic search strategy. Bootstrapping was performed within PAUP*. The tree was initially displayed in Treeview version 1.6.6. (http://taxonomy.zoology.gla.ac.uk/rod/treeview.html), then opened in NJPlot (http://pbil.univ-lyon1.fr/software/njplot.html), and bootstrap values added to the significant (bootstrap values >70) branches by using Microsoft Word. Pairwise comparison of the NSP1 and E1 sequences obtained at PWH by using BioEdit were identical (i.e., 100% similarity) to the corresponding sequences in the full-length chikungunya genome sequence (LR2006_OPY1_Reunion_2006) deposited in GenBank (accession no. DQ443544) from the same outbreak in La Reunion.

Figure A1 shows the phylogenetic tree for chikungunya E1 sequences (199 bp) constructed by using PAUP* under a Tamura-Nei 1993 model with invariable sites (i.e., TrN+I), as selected by MODELTEST (version 3.7) under the Akaike information criteria (AIC). The bracket shows that the PWH patient E1 sequence (DQ489787) clusters most closely with the outbreak La Reunion sequence (LR2006_OPY1_Reunion_2006_E1, DQ443544). Only bootstrap values >70 are shown and considered significant. Figure A2 shows the phylogenetic tree for chikungunya NSP1 sequences (314 bp), constructed using PAUP* under a transitional model with equal base frequencies with a γ-distributed rate of substitution (i.e., TIM+G), as selected by MODELTEST (version 3.7) under the AIC. The bracket shows that the PWH patient NSP1 sequence (GenBank accession no. DQ489788) clusters most closely with the La Reunion chikungunya outbreak sequence (LR2006_OPY1_Reunion_2006_NSP1, DQ443544).

**Appendix References**

1. Wong CK, Lam CW, Wu AK, Ip WK, Lee NL, Chan IH, et al. Plasma inflammatory cytokines and chemokines in severe acute respiratory syndrome. Clin Exp Immunol. 2004;136:95–103.

2. Tang NL, Chan PK, Wong CK, To KF, Wu AK, Sung YM, et al. Early enhanced expression of interferon-inducible protein-10 (CXCL-10) and other chemokines predicts adverse outcome in severe acute respiratory syndrome. Clin Chem. 2005;51:2333–40.

3. Pastorino B, Muyembe-Tamfum JJ, Bessaud M, Tock F, Tolou H, Durand JP, et al. Epidemic resurgence of Chikungunya virus in democratic Republic of the Congo: identification of a new central African strain. J Med Virol. 2004;74:277–82.

4. Hasebe F, Parquet MC, Pandey BD, Mathenge EG, Morita K, Balasubramaniam V, et al. Combined detection and genotyping of Chikungunya virus by a specific reverse transcription-polymerase chain reaction. J Med Virol. 2002;67:370–4.

5. Thompson JD, Gibson TJ, Plewniak F, Jeanmougin F, Higgins DG. The ClustalX windows interface: flexible strategies for multiple sequence alignment aided by quality analysis tools. Nucleic Acids Res 1997;25:4876–82.

6. Swofford DL. PAUP*. Phylogenetic analysis using parsimony (*andother methods). Version 4. Sunderland (MA): Sinauer Associates;2001. Available from http://paup.csit.fsu.edu/win.html

7. Posada D, Crandall KA. MODELTEST: testing the model of DNA substitution. Bioinformatics. 1998;14:817–8. Available from http://darwin.uvigo.es/software/modeltest.html
